# Incidence of altered proteins in the aging brain: Implications for biological diagnostic markers

**DOI:** 10.1177/13872877251414978

**Published:** 2026-02-04

**Authors:** Irina Alafuzoff, Sylwia Libard

**Affiliations:** 1 Department of Pathology, Uppsala University Hospital, Uppsala, Sweden; 2 Department of Immunology, Genetics and Pathology, Uppsala University, Uppsala, Sweden

**Keywords:** aging, α-synuclein, alzheimer's disease, amyloid-β protein, biomarker relevance, cognitive decline, hyperphosphorylated τ, mixed proteinopathies, transactive DNA-binding protein 43

## Abstract

**Background:**

In aging-related neurodegeneration, therapeutic interventions directed at defined protein targets have been launched. A key step has been developing diagnostic biological markers, followed by immunotherapy. These steps have been achieved for amyloid-β protein (Aβ).

**Objective:**

To evaluate, based on brain pathology, how frequently Aβ would be detectable in blood or cerebrospinal fluid in older individuals.

**Methods:**

We assessed brain tissue from 1825 deceased subjects, 20% of whom were demented. We examined the presence of Aβ, hyperphosphorylated τ (HPτ), Transactive DNA-binding protein 43 (TDP-43), and α-synuclein (αS) using immunohistochemistry. The extent of these alterations was assessed following current consensus criteria.

**Results:**

The combination of Aβ/HPτ, constituting Alzheimer's disease neuropathological change (ADNC), was detected in 64% of subjects, increasing significantly (Pearson's Chi-Square *p* = 0.001) from 46% in the 5^th^ decade to 81% in the 9^th^ decade. In 506 subjects (28% of the cohort), intermediate or high levels of ADNC were observed an extent reported as assessable in cerebrospinal fluid. Among these, 235 were non-demented and would have been identified as being at risk of Alzheimer's disease. Most (74%) displayed concomitant pathologies, making it impossible at this stage to determine which pathology will eventually lead to cognitive impairment.

**Conclusions:**

Common ADNC and frequent concomitant pathologies in older individuals will influence the interpretation of diagnostic biological tests. Current tests can confirm that a subject displays ADNC; however, a definite diagnosis can only be achieved through postmortem neuropathological assessment. Present diagnostic tests are too crude to detect early or low-level ADNC.

## Introduction

Aging-related neurodegeneration leading to cognitive impairment is a major social and economic challenge. The most common clinical diagnosis associated with aging-related cognitive impairment is Alzheimer's disease (AD).^1^ The main pathological hallmarks observed in the brains of patients with AD are amyloid-β protein (Aβ) and hyperphosphorylated τ (HPτ), alterations historically identified as tangles and plaques using histochemical silver staining since the disease was first defined.^2–5^ Current praxis has relied on postmortem (PM) analysis of the brain to establish a definitive diagnosis by assessing the extent of AD neuropathologic change (ADNC) based on the regional distribution of Aβ and HPτ.^6,7^ Diagnostics for aging-related neurodegeneration and AD have evolved significantly in recent years.

It has been evident for approximately 20 years that, in addition to ADNC, aged individuals and subjects with cognitive impairment often display concomitant altered proteins in the brain, such as α-synuclein (αS) and Transactive DNA-binding protein 43 (TDP-43).^8–12^ These protein alterations also affect brain function; in 2021, McAleese and colleagues, in their assessment of 670 brains, reported that concomitant pathologies contribute to the transition from mild cognitive impairment to dementia.^13^ In line with ADNC, the extent of both αS and TDP-43 pathology is assessed by analyzing their regional distribution, given as a stage of pathology.^14,15^

In parallel with these developments, there has been an intensive search for biological diagnostic markers in cerebrospinal fluid (CSF) and blood.^16–18^ Protein alterations arising in the brain can be detected in CSF or blood, forming the basis of such markers. This research has been facilitated by the need for more precise *in vivo* diagnostics. Two common hallmark lesions, Aβ and HPτ, have long been assessed in CSF, and analyses in blood samples are now ubderway^19,20^ In addition, there are ongoing efforts to assess αS in both CSF and blood samples.^21–23^

This emphasizes the urgent need to increase our knowledge regarding the pathology observed in the brains of older individuals, particularly those who are cognitively unimpaired.

The use of biological diagnostic markers is also of significance in the planning and implementation of targeted precision medicine. In line with this, pharmacological treatment directed at one of the hallmark lesions, Aβ, has already been initiated.^24^ It has been suggested that, for such treatment to be effective, the altered protein Aβ must be targeted at an early stage of the disease process.^24^

Here, we assessed the incidence of common altered proteins, HPτ, Aβ, TDP43, and αS, in a large PM cohort, independent of the subjects’ clinical presentation. The proteins detected in the brain would presumably also be detectable in CSF or blood using sensitive, currently available and emerging analytical methods. The primary objective was to estimate what the outcomes might have been if CSF or blood had been analyzed for these protein alterations prior to death. Such testing would aim to identify individuals at an early stage of neurodegeneration who are therefore at risk of developing a severe disease over time. The use of such tests forms the basis of precision medicine, in which a defined pathological culprit is specifically targeted.

## Methods

### Participants

The study cohort consisted of 1825 subjects who, prior to death, had either lived at home, resided in a long-term care facility, been admitted to a hospital shortly before death, or received care at a regional hospital or at Uppsala University Hospital. The ages at death ranged from 50 to 102 years. Of the total cohort, 1038 (57%) were male and 787 were female. The flowchart (Table 1) outlines the overall processing of the cohort.

**Table 1.** Flowchart.

1825 subjects, mean age at death 76 years (standard error of means ± 0.2) subjects referred for autopsy including a neuropathological assessment			
clinical data were pulled out from the referral and medical records			
372 displayed dementia during life 80 ± 0.4 years	176 various neurological complains 75 ± 0.7 years	343 Stroke or TIA 76 ± 0.5 years	934 neurologically unimpaired 75 ± 0.3 years
Clinical diagnosis: dementia not otherwise specified (dNOS), Alzheimer's Disease (AD), Dementia with Lewy bodies (DLB), Parkinson's Disease with Dementia (PDD), Multi System Atrophy with dementia, Frontotemporal dementia, Amyotrophic Lateral Sclerosis with dementia, Korsakoff's syndrome and Vascular Dementia			
Immunohistochemical stain to assess regional distribution of Aβ, HPτ,αS and TDP43 as given in material and methods			
for the extent of Aβ, Thal phase ^26,34^, for extent of HPτ, Braak stage ^25^ for αS, Braak stage^14,33^ and for TDP43 Josephs stage^15^ were assessed			
When primary tauopathy was suspected 4R and 3Rtau stains were carried out as given in material and methods			
Diagnosis of primary tauopathies and TDP43-pathies followed current published criteria ADNC^6,7^; LBD^34,35^; PSP^41,42^ AGD^43^; PART^37^; TpD^38,39^; FTLD^44^; LATE-NC.^36^			

Neurological complains: multiple sclerosis, epilepsy, depression, various psychiatric diseases, various deficits due to excessive alcohol consumption; TIA: transient ischemic attack; Aβ: amyloid-β protein; HPτ: Hyperphosphorylated τ; αS: α-synuclein; TDP43: transactive DNA binding protein 43; 4Rtau: 4 repeat tau; 3Rtau: 3 repeat tau; ADNC: Alzheimer's Disease Neuropathologic Change; LBD: Lewy body diseases; PSP: progressive supranuclear palsy; AGD: argyrophilic grain disease; PART: primary age related tauopathy; TpD: Tangle predominant dementia; FTLD: frontotemporal lobar degeneration; LATE-NC: limbic-predominant age-related TDP encephalopathy - neuropathologic change.

Clinical data regarding dementia or neurological deficits were obtained from referrals. In most dementia cases, the diagnosis had been made by a primary healthcare provider. Evaluation by a general practitioner typically includes a clinical assessment, interviews with relatives, and basic blood tests. CSF analysis is primarily carried out in younger subjects (<65 years) displaying symptoms, or in those with unusual clinical presentations. Among the 1825 subjects, 372 individuals (20%) had shown symptoms of dementia during life. Thus, based on medical records, 1463 subjects had not been diagnosed with dementia. Of these, 934 (64%) were considered neurologically unimpaired. A further 176 subjects had experienced various neurological conditions, including depression, epilepsy, Parkinson's disease (PD), multiple sclerosis, amyotrophic lateral sclerosis, ataxia, psychiatric diseases, or neurological deficits due to excessive alcohol consumption. Furthermore, 343 subjects had suffered a stroke or transient ischemic attack (TIA). Some of these 1463 subjects may nonetheless have displayed mild cognitive impairment.

The subjects were referred for autopsy by a healthcare provider, primarily to determine the cause of death. Many had not required continuous medical care, and the cause of death could therefore not be established without an autopsy. The study of postmortem was approved by the local ethics committee (Dnr 2011/286 and Dnr 2020/4184).

### Tissue processing

At autopsy, the brains were removed and placed in buffered formalin for one week. Thereafter, they were sectioned, and at least 16 tissue samples were collected for histological and immunohistochemical (IHC) staining. The neuroanatomical regions sampled included the frontal, temporal, parietal, occipital and motor cortices; gyrus cinguli; anterior and posterior hippocampus; basal forebrain including the amygdala; striatum; thalamus; mesencephalon including the substantia nigra; pons including the locus coeruleus; medulla including the motor nucleus of the vagus; vermis; and cerebellar hemisphere. The samples were placed in commercial mega-cassettes for further fixation in 4% buffered formalin. After an additional two weeks of fixation, the automatic paraffin-embedding procedure was initiated.

All sections, 7 μm thick, were stained with hematoxylin-eosin (HE) stain. Eventual vascular lesions, including infarcts, were registered in all 16 sections. Following IHC stain in Autostainer Link 48 was carried out to visualize the altered proteins Aβ, HPτ, αS, and TDP-43. The proteins were assessed in predefined regions, as each protein has a characteristic initiation site and predictable progression in the brain.^14,15,25,26^ For Aβ/Dako, clone 6F/3D was used in a dilution 1:100, pre-treatment 80% Formic acid for 2 min. Aβ is first seen in the neocortex and progresses through central regions of the brain to the cerebellum; it was therefore assessed in at least the parietal cortex and basal forebrain, including the amygdala. For HPτ/Thermo Scientific, clone AT8, was used in dilution 1:500. HPτ initially appears in the locus coeruleus at the level of the pons and/or the hippocampus and progresses towards the neocortex. It was assessed at least in the pons, hippocampus and basal forebrain, including the amygdala. For αS/NovoCastra, clone KM51, was used in a dilution 1:100, pre-treatment with Citrate buffer pH 6.0 and 80% Formic acid for 2 min. αS is first observed in the motor nucleus of the vagus in the medulla and progresses to the neocortex via the pons and midbrain. It was assessed at least in the medulla, midbrain and basal forebrain, including the amygdala. For TDP43/Cosmo Bio, clone 11-9, was used in a dilution 1:100, pre-treatment with Citrate buffer pH 6.0.

TDP-43 is seen primarily in the amygdala of aged subjects and was assessed at least in the basal forebrain, including the amygdala and hippocampus. Thus, in total, initially at least two brain sections were assessed for Aβ and TDP-43, and three for HPτ and αS. If these screening sections were positive, staining was carried out on additional sections. Some studies suggest that the olfactory bulb is affected early by HPτ and αS pathology in neurodegenerative diseases.^27^ This is in line with the common early clinical symptom of loss of smell observed in subjects with cognitive impairment.^28^ The olfactory bulb was not sampled in all 1825 cases and was therefore not assessed. Its absence may mean that some very early-stage cases were missed. The antibodies and staining protocols used here have yielded reproducible results in multi-center studies carried out by BrainNet Europe.^29–31^

### Neuropathological assessment and diagnosis

Staging of protein alterations observed in IHC-stained sections was based on current consensus criteria, i.e., Braak stage for HPτ, Thal phase for Aβ, Josephs stage for TDP43, and Braak stage for αS.^14,15,25,26^ The assessment strategies used have yielded reproducible results in multi-center studies carried out by BrainNet Europe.^32–34^ The BrainNet Europe strategy for αS is based on a dichotomous evaluation of labelled lesions, an approach recently confirmed by Attems and colleagues in a multi-center study.^35^ The Josephs staging system was implemented for the diagnosis of LATE-NC.^36^

For the diagnosis of Primary age-related tauopathy (PART), the criteria described by Crary and colleagues were followed, with the exception that none of our PART cases displayed any Aβ pathology.^37^ For tangle-predominant dementia (TpD), publications by Bancher and Jellinger, and Yamada, were consulted.^38,39^ If a primary tauopathy, such as progressive supranuclear palsy (PSP), corticobasal degeneration (CBD), or argyrophilic grain disease (AGD), was suspected based on HPτ staining or clinical data, IHC staining with antibodies specific to 4Rtau (4Rtau/Millipore, clone 1E1-A6, dilution 1:200, pre-treatment with Citrate buffer pH 6.0 and 80% Formic acid for 10 min) and 3Rtau (3Rtau/Millipore, clone 8E6C, pre-treatment pressure cooker in distilled H_2_O and 80% Formic Acid for 30 min) was performed.^40^

For the diagnosis of PSP, the Rainwater Charitable Foundation criteria were applied, and for AGD, the diagnostic criteria described by Heiko and Eva Braak were followed.^42,43^ For the diagnosis of frontotemporal lobar degeneration (FTLD) and limbic-predominant Age-related TDP encephalopathy (LATE), recently published criteria were followed.^36,44^

### Statistical analysis

For statistical analysis, IBM SPSS was used, applying non-parametric tests. For descriptive statistics, the mean ± Standard Error of the mean (m ± SE) was presented. Statistical differences between the studied groups were assessed using the non-parametric Kruskal Wallis (KW) and Mann-Whitney U (MWU) tests. For the contingency of categorical data, Fisher's exact (FE) test and Pearson's Chi-Square (PCS) test were applied.

## Results

### Clinical summary

The study included 1825 subjects, with a mean age at death of 76 ± 0.2 years. Females (77 ± 0.1 years at death) were significantly (MWU = 0.008) older at the time of death than males (75 ± 0.1 years at death). Dementia was observed in 20% of the subjects, with a significantly (FE < 0.001) higher prevalence in females compared to males (25% versus 17%, respectively). Among those with dementia, the mean age at death for female subjects (80 ± 0.1 years at death) was significantly higher (MWU = 0.000) than that of males (75 ± 0.1 years at death). In most cases, the clinical diagnosis of dementia was dementia not otherwise specified (dNOS), accounting for 65% of cases (mean age at death 81 ± 0.5 years), followed by AD in 20% (80 ± 1.1 years) and by dementia with Lewy bodies/PD with dementia (DLB/PDD) or dementia associated with multi-system atrophy in 9% (78 ± 1.1 years). Less common clinical diagnoses included frontotemporal dementia (FTD) in 4% (71 ± 2.4 years), PSP/CBD in 1% (78 ± 2.0 years), vascular dementia (VaD) in 1% (87 ± 2.0 years), and dementia caused by excessive alcohol consumption, including Korsakoff's Syndrome, in 1% (69 ± 3.5 years). The mean age at death differed significantly between groups (KW *p* < 0.001), with the youngest being those with clinical diagnosis of Korsakoff's Syndrome and the oldest being those with clinical diagnosis of VaD.

### Protein alterations

The incidence of altered proteins in the brains of the subjects, as well as the incidence of various combinations of protein alterations, is summarized in Tables 2–4. Table 2 presents the results for the whole cohort in relation to age, Table 3 shows the cohort divided by clinical signs of dementia and age, and Table 4 presents data based on the clinical diagnosis.

**Table 2.** The percentage of subjects with altered proteins and their various combinations among 1825 subjects, categorized by age groups.

Alteration	number (percent)	AGE AT DEATH IN YEARS					PCS
		50–59	60–69	70–79	80–89	≥90	
all subjects, number	1825	107	347	683	549	139	
males / females, number	1038/787	70/37	211/136	384/299	303/246	70/69	
with dementia	372 (20)	7	8	19	31	30	<0.001
demented males / females, number	176/196	7/0	10/18	55/72	86/83	18/23	
with Hyperphosphorylated τ (HPτ)	1791 (98)	95	95	99	100	100	<0.001
with Amyloid β-protein (Aβ)	1169 (64)	47	52	61	75	81	<0.001
with Transactive DNA binding protein 43 (TDP)	660 (36)	10	20	32	49	66	<0.001
with α-synuclein αS	393 (22)	8	13	22	28	27	<0.001
no protein alteration	23 (1)	4	4	1	<1		<0.001
only HPτ	422 (23)	42	35	26	12	6	<0.001
only Aβ	10 (1)	1	1	<1	<1		
only αS	1 (<1)		0.3				
with only HPτ & Aβ	506 (28)	36	35	28	25	21	0.017
with HPτ & TDP	112 (6)	2	2	6	7	9	
with HPτ & αS	68 (4)	6	10	4	4	2	
with HPτ & Aβ & TDP	359 (20)	8	6	17	28	37	<0.001
with HPτ & Aβ & αS	136 (8)	2	1	8	10	5	0.017
with Aβ & TDP & αS	30 (2)			2	2	2	
with HPτ & Aβ & TDP & αS	158 (9)	1	3	8	13	18	<0.001
all with HPτ & Aβ	1159 (64)	46	51	60	75	81	<0.001

PCS: Pearson's Chi-Squared test.

**Table 3.** The percentage of subjects with altered proteins and their various combinations among in 1453 non-demented and 372 demented subjects, categorized by age groups.

Alteration	number (percent)	FE	AGE AT DEATH IN YEARS					PCS
			50-59	60-69	70-79	80-89	≥90	
**all non-demented subjects, number**	**1453**		**100**	**319**	**556**	**380**	**98**	
with HPτ	1420 (98)		95	94	99	100	100	<0.001
with Aβ	850 (59)		44	51	57	68	75	<0.001
with TDP43	413 (28)		8	15	26	41	61	<0.001
with αS	259 (18)		7	12	18	26	19	<0.001
no protein alteration	22 (2)		4	4	1	<1		<0.001
only HPτ	409 (28)		45	35	30	18	8	<0.001
only Aβ	10 (2)		1	1	1	<1		
only αS	1 (<1)			<1				
with only HPτ & Aβ	438 (30)		35	33	31	27	24	
with HPτ & TDP	92 (7)		2	3	7	8	13	<0.001
with HPτ & αS	58 (4)		5	3	5	5	2	
with HPτ & Aβ & TDP	223(15)		6	9	13	21	36	< 0.001
with HPτ & Aβ & αS	103 (7)		2	6	7	10	5	
with Aβ & TDP & αS	21 (1)			1	1	2	3	
with HPτ & Aβ & TDP & αS	76 (5)			1	5	10	9	<0.001
all with HPτ & Aβ	840 (58)		43	49	56	67	73	<0.001
**all demented subjects**	**372**		**7**	**28**	**127**	**169**	**41**	
with HPτ	371 (100)	0,04	100	100	99.2	100	100	
with Aβ	319 (86)	< 0.001	86	61	76	90	90	<0.001
with TDP43	247 (66)	<0.001	43	75	61	68	78	
with αS	134 (36)	< 0.001	29	25	39	33	46	
no protein alteration	1 (<1)	0.036			1			
only HPτ	13(4)	<0.001		4	9	1		0.003
with only HPτ & Aβ	68 (18)	<0.001	43	21	17	19	15	
with HPτ & TDP	20 (5)			29	5	4		<0.001
with HPτ & αS	10 (3)		14		3	2	2	
with HPτ & Aβ & TDP	136 (37)	< 0.001	29	21	31	44	39	0.069
with HPτ & Aβ & αS	33 (9)				9	11	5	
with Aβ & TDP & αS	9 (2)				6	1		0.087
with HPτ & Aβ & TDP & αS	82 (22)	< 0.001	14	25	21	19	39	0.078
all with HPτ & Aβ	319 (86)	< 0.001	86	68	77	92	98	<0.001

** **HPτ, Hyperphosphorylated τ; Aβ, Amyloid β-protein; TDP43, Transactive DNA binding protein 43; αS, α-synuclein; FE, Fisher’s exact test; significant difference when comparing 1453 non-demented with 372 demented subjects; PCS, Pearson’s Chi-Squared test

**Table 4.** The percentage of subjects with altered proteins and their various combinations among 1453 non-demented subjects, categorized by clinical groups.

Alteration	non-demented number (%)	neurologically unimpaired	Various neurological complains	history of stroke or TIA	PCS
all subjects, number	1453	934	176	343	
mean age at death ± standard error of means	75 ± 0.3	75 ± 0.3	75 ± 0.7	76 ± 0.5	
males / females, percent	59/41	60/40	49/51	62/38	
with Hyperphosphorylated τ (HPτ)	1420 (98)	97	98	99	0.032
with Amyloid β-protein (Aβ)	850 (59)	61	57	52	0.008
with Transactive DNA binding protein 43 (TDP)	413 (28)	31	22	25	0.009
with α-synuclein αS	259 (18)	15	30	19	<0.001
no protein alteration	22 (2)	2	2	<1	
only HPτ	409 (28)	26	24	36	<0.001
only Aβ	10 (2)	1		<1	
only αS	1 (<1)	<1			
with only HPτ & Aβ	438 (30)	32	31	26	
with HPτ & TDP	92 (7)	7	5	6	
with HPτ & αS	58 (4)	3	10	5	<0.001
with HPτ & Aβ & TDP	223 (15)	18	9	13	0.003
with HPτ & Aβ & αS	103 (7)	6	13	8	0.004
with Aβ & TDP & αS	21 (1)	1	3	2	
with HPτ & Aβ & TDP & αS	76 (5)	6	5	5	
all with HPτ & Aβ	840 (58)	60	57	51	0.016

TIA: transient ischemic attack; PCS: Pearson's Chi-Squared test carried out between clinical categories.

There were 23 (1%) subjects who lacked detectable protein alterations in the brain (mean age at death 66.3 ± 0.5), including 11 males and 12 females. Among these, one individual, a 70-year-old male, had displayed signs of dementia during life (clinical diagnosis dNOS). Neuropathological examination indicated that his cognitive impairment was attributable to substantial vascular brain lesions observed in HE-stained sections, consistent with a diagnosis of VaD.

HPτ pathology was observed in 98% of all subjects (Table 2). There were 422 subjects with only HPτ pathology, with a mean age at death of 72 ± 0.1 years; this group included 257 males (71 ± 0.2 years) and 165 females (73 ± 0.3 years), most of whom displayed features consistent with PART. In most cases, HPτ pathology was relatively sparse. Thirteen subjects (3%) with only HPτ pathology were demented (75 ± 0.6 years at death), including eight females and seven males. Among these, eight subjects displayed pathology in line with PSP (clinical diagnoses: 4× dNOS, 2× AD, 2× PSP), one displayed AGD (clinical diagnosis: Korsakoff's Syndrome), and one displayed pathology consistent with TpD (clinical diagnosis: dNOS). In three of the demented subjects, HPτ pathology (PART) coexisted with substantial vascular brain alterations, consistent with VaD (clinical diagnosis: dNOS).

Ten subjects (mean age at death 69 ± 0.7 years) exhibited only Aβ pathology. This group included six males and four females, none of whom displayed signs of dementia during life. Five of these 10 subjects also displayed cerebral amyloid angiopathy (CAA) in addition to parenchyma Aβ.

αS pathology was observed in 393 subjects (22%). Among these, one subject, a 63-year-old male, who was clinically unaffected, displayed only αS pathology.

TDP-43 pathology was observed in 660 subjects (36%) and was never seen in isolation; it always occurred alongside another protein alteration. In 37 subjects (6%), the distribution of TDP-43 pathology was consistent with a diagnosis of FTLD, and 24 of these were demented (clinical diagnosis: 14×dNOS, 9×FTD, 1×PSP), while the majority (76%) exhibited features of LATE.

Among the remaining 1369 subjects, various combinations of HPτ, Aβ, TDP43, and αS were noted (Tables 2–4).

The most common protein combination (63.5%) observed was HPτ/Aβ, and this combination increased significantly with age, from 46% to 81% (PCS *p* < 0.001) (Table 2). Pure HPτ/Aβ pathology was seen in 28% of subjects, decreasing significantly with age (PCS *p* < 0.017) (Table 2).

There were 1453 subjects who did not display dementia during life (mean age at death 74.6 ± 0.3 years); of these, 59% were male (Table 3). In this group, HPτ/Aβ was the most common combination (58%), increasing significantly with age, from 43% to 73% (PSC *p* < 0.001). Conversely, pure HPτ/Aβ pathology was seen in 30% of these subjects and decreased with age, from 35% to 24% (Table 3).

There were 372 subjects with dementia (mean age at death 80 ± 0.4 years), 53% of whom were female (Table 3). In this group, HPτ/Aβ was the most common combination, observed in 86% of subjects, and increased significantly with age, from 86% to 98% (PCS *p* < 0.001). Conversely, pure HPτ/Aβ pathology was seen in 18% of subjects and decreased with age, from 43% to 15%. The age-related decreases and increases in various combinations of altered proteins in the brain are summarized in Table 3.

Table 4 presents outcomes in relation to clinical diagnosis. Significant differences were noted among the three non-demented groups: neurologically unimpaired subjects, those with various neurologic complaints, and those with a history of TIA or Stroke. Some of the differences were related to αS, likely reflecting the presence of subjects with PD in the second group. Additionally, in the stroke/TIA group, the incidence of HPτ/Aβ pathology was lower compared to other non-demented groups, whereas the number of subjects with HPτ pathology alone was significantly higher (PCS *p* < 0.001).

The extent of pathologies at moderate or higher levels, i.e., Aβ Thal phase ≥ 3, Braak HPτ stage ≥3, αS Braak stage ≥3 and TDP-43 Josephs stage ≥2, is summarized in Table 5. These levels would probably have been detectable by imaging or analysis of CSF and/or blood if sensitive methods had been applied. Among non-demented subjects, 28% would have displayed moderate to high levels of Aβ and 21% moderate to high levels of HPτ.

**Table 5.** Number and percent of subjects with moderate extent of altered proteins in their brain. Moderate extent of altered proteins based on regional distribution.

	Moderate extent	All cases	Demented	Non-demented
number		1825	373	1453
male / females (percent)		1038(57) / 787(43)	176 (47) 196 (53)	862 (59) / 591 (41)
mean age ± standard error of means		76 ± 0.2	80 ± 0.4	75 ± 0.3
with hyperphosphorylated τ	Braak stage ≥3^25^	588 (32)	288 (77)	300 (21)
with amyloid-β protein	Thal phase ≥ 3^26,34^	671 (37)	270 (73)	401 (28)
with α synuclein	Braak stage ≥ 3^14,33^	339 (19)	127 (34)	212 (15)
with transactive DNA binding protein 43	Josephs stage ≥ 2^15^	450 (25)	180 (48)	270 (19)

Overall, 1076 subjects displayed ADNC, of whom 27% were demented. Of these, 570 subjects displayed low levels of ADNC, independent of concomitant pathologies; 60% were male, with a mean age at death of 74 ± 0.4 years, and 23 (4%) were demented. Intermediate levels of ADNC was observed for 390 subjects, 53% of whom were male, with a mean age at death of 82 ± 0.4 years; 155 (40%) of these subjects were demented.

Thus, 235 non-demented subjects displayed intermediate ADNC (60% of all subjects with intermediate ADNC) and would probably have been diagnosed with AD if imaging or CSF/blood sample analysis had been performed. Finally, 116 subjects displayed high levels of ADNC; 41% were male, with a mean age at death of 80 ± 0.8 years, and all of these subjects were demented.

Table 6 summarizes the incidence of concomitant pathologies in subjects with intermediate and high levels of ADNC. The number of subjects with all four protein alterations was significantly higher in demented compared with non-demented individuals (PCS *p* = 0.003). Comparisons of non-demented subjects across the various clinical groups should be interpreted with caution, due to the limited number of subjects in two of the three groups.

**Table 6.** Altered proteins and their combinations in subjects with intermediate (non-demented and demented) and high level (only demented) of Alzheimer's disease neuropathologic change.^6,7^

Alteration	Demented		Non-demented	Neurologically unimpaired	Various neurological complains	History of stroke or TIA
	Intermediate	High	Number (%)	Percent	Percent	Percent
all subjects	155	116	235	155	27	53
males / females	80/75	47/69	125/110	85/70	11/16	29/24
mean age at death ± SE	82±0.8	80±0.9	81±0.5	81±0.7	79.7±1.4	82±1.1
with only HPτ & Aβ	25	16	26	26	33	25
with HPτ & Aβ & TDP	41	42	46	52^2^	19^25^	43^5^
with HPτ & Aβ & αS	10	8	10	5^1,4^	26^1^	17^4^
with HPτ & Aβ & TDP & αS	24^3^	34^3^	17^3^	17	22	15

SE: standard error of means; HPτ: hyperphosphorylated τ; Aβ: amyloid-β protein; TDP43: transactive DNA binding protein 43; αA: α-synuclein; TIA: transient ischemic attack; PCS: Pearson's Chi-Squared test. ^1^ < 0.001, ^2^0.001, ^3^0.003, ^4^0.007, ^5^0.03.

## Discussion

Here, using a large population-based cohort including 1825 subjects, including 1453 cognitively unimpaired and 373 demented individuals, and applying sensitive IHC methods, we demonstrate that a vast number of subjects display HPτ in combination with Aβ, i.e., ADNC, in their brain. This observation is of significance, as it suggests that in 64% of all subjects, and in 58% of cognitively unimpaired subjects, sensitive biological diagnostic markers would suggest that they either have or are at risk of developing AD.

Currently, three biological diagnostic markers are of particular interest, i.e., positron emission tomography (PET), CSF analysis, and blood-based analysis of Aβ and HPτ.

PET analysis has been used relatively frequently in recent years, and numerous reports, including those discussing its limitations when Aβ aggregates have specific characteristics (for example, cotton wool plaques) are available.^45^ Overall, the performance of Aβ PET is, based on many studies, favorable for distinguishing cognitively unimpaired subjects from those who are demented.^46^ However, the method is less effective at identifying subjects with mild cognitive impairment, who are of interest for therapeutic intervention.^46^

In 2016, it was reported that CSF analysis can detect cerebral Aβ earlier than PET, and subsequent studies showed a correlation between CSF-Aβ_42_ and PiB-PET when collected within six years of each other.^47,48^ Contradictory reports suggesting that PET is more sensitive have also been published.^49^ In general, CSF analysis seems to be more sensitive than imaging.^50^ However, lumbar puncture is invasive and thus not commonly performed in cognitively impaired subjects; it is primarily used in younger subjects or those with unusual clinical presentations.

The third diagnostic marker, based on blood, has generated major interest. In 2022, a study of 427 subjects reported that CSF Aβ_42/_Aβ_40_ predicts Aβ PET burden up to relatively high levels, whereas plasma Aβ_42/_Aβ_40_ predicts Aβ PET burden only up to lover levels.^51^ Recently, it has been reported that plasma biomarkers can detect early AD, confirming findings first published in 2019 by Pérez-Grijalba and colleagues.^52,53^ These authors also noted that plasma biomarkers correlate well with CSF biomarker outcomes. Taken together, recent publications suggest that PiB-PET, CSF Aβ_42/_Aβ_40,_ and plasma Aβ_42/_Aβ_40_ perform quite equally. In parallel with the above, some reports suggest that plasma HPτ may be more effective at identifying Aβ positive individuals at an early stage, and thus may be particularly suitable for selecting participants for Aβ-targeted trials.^54,55^ This aligns with the study from 2011 by Braak and colleagues, who assessed the brains of 2332 subjects aged 0 to 100 years at death and concluded that HPτ pathology proceeds Aβ pathology.^56^

In 2022, a study of 101 subjects (16 with AD, 43 with primary tauopathy, 27 with FTLD and 15 with other diagnoses) reported that CSF HPτ/Aβ_42_ and Aβ_42_/Aβ_40_ demonstrated high sensitivity, specificity and overall diagnostic performance for detecting intermediate to high levels of ADNC.^57^ Although the number of AD cases in the referred study is relatively low, the results suggest that the extent of pathology must reach at least an intermediate level in the brain before it can be reliably detected in CSF. This further implies that intermediate to high levels of ADNC are required when current diagnostic markers are used: PET, CSF or blood.

These biological diagnostic markers can certainly be used to confirm that clinically observed cognitive impairment may be caused by underlying ADNC. However, they are unlikely to be sufficiently sensitive to identify subjects at an early stage of the disease, i.e., those with low rather than intermediate to high levels of ADNC.

Thus, there are differences in the performance of the available biological diagnostic markers. CSF-and blood-Aβ measurements seem to be closely aligned with PiB-PET, detecting brain alterations only once they have reached an extent corresponding to intermediate to high levels of ADNC.

Based on the above, not all of our 1076 subjects with ADNC in our cohort would have been detected using the currently available diagnostic analyses. Of these individuals, 390 subjects had intermediate-level ADNC and 116 had high-level ADNC. These 506 subjects would likely have been diagnosed with AD, or considered at risk of developing AD, had a biological diagnostic test been performed during life. Noteworthy, 235 of the subjects with intermediate-level ADNC were non-demented. The question arises: should these subjects be informed that they are at risk of developing AD? Can we be certain that progression is inevitable, and that any future cognitive impairment would be caused by ADNC rather than by concomitant alterations? Moreover, should they be selected for therapeutic intervention, or would this be too late, given that intermediate-level ADNC already reflects a substantial pathological burden?

Concerns regarding the use of biological diagnostic markers have already been raised by Dubois and colleagues (2021), who emphasized the need to distinguish between biomarker-positive subjects with an AD phenotype and biomarker-positive cognitively unimpaired subjects.^58^

Another major limitation of using biological diagnostic markers as diagnostic tools is the frequent presence of concomitant pathologies. Recently, Kapasi and colleagues reported, while assessing 428 older subjects (mean age 91 years), that only 26% displayed pure ADNC, i.e., only HPτ and Aβ. Similarly, in 2023, White and colleagues found that only 20% of their 387 demented subjects displayed pure ADNC.^10,59^

Our observations are consistent with the previous reports: only 28% of the whole cohort displayed pure ADNC (30% of non-demented; 18% of demented subjects). Noteworthy, our cohort was younger (mean age 76 years) compared to the cohort of Kapasi and colleagues, and it included various clinical presentations, making direct comparison difficult. Furthermore, comparing concomitant pathologies across studies is somewhat problematic as the mode of reporting differs. Both Kapasi and White included vascular alterations as a type of concomitant pathology, whereas we did not.^10,59^ Despite the difference, the overall results are agreeable. Kapasi reported ≥1 concomitant pathologies in 68% of cases, while White reported one additional concomitant pathology in 25% and two in 11% of their cohort. In our study, among non-demented subjects, ADNC accompanied by one additional protein alteration was observed in 22%, and by two additional alterations in 5%. Among demented subjects, ADNC with one additional pathology was seen in 46%, and with two in 22% of the cases. In conclusion, concomitant pathology is frequently seen in most subjects with ADNC, and its presence is likely to affect responses to potential therapeutic interventions.

Noteworthy, concomitant protein alterations were also seen in subjects with intermediate levels of ADNC the level of brain pathology that is detectable using current biological diagnostic tests.^57^ Among our subjects with intermediate ADNC, pure ADNC was observed in only 16 to 33%, and a significantly higher proportion of demented subjects displayed both TDP-43 and αS compared with non-demented subjects. As demonstrated here, it would be impossible to assign a “definite” diagnosis based solely on biological diagnostic markers, and it is equally challenging to predict the evolution of the clinical phenotype given the frequent occurrence of concomitant pathology.

The question remains: should the 235 cognitively unimpaired subjects who would be identified as being at risk of AD through biological diagnostic markers be considered for Aβ immunotherapy? Current evidence suggests that treatment should be initiated at an early stage, as only early intervention is presumed to be beneficial.^24^ However, whether the intermediate-level of ADNC qualifies as “early enough” is doubtful, as HPτ pathology has already reached the temporo-occipital cortices by this stage.

Moreover, the proportion of subjects with pure ADNC, i.e., without concomitant protein alterations such as TDP-43 and/or αS, was low, at only 26%. This finding may indicate that only this subset of subjects could benefit, at least to some extent, from Aβ immunotherapy. This presumption, based on brain pathology, is in line with recent clinical outcomes indicating that a clinically meaningful benefit is observed in only approximately 27 to 35% of treated subjects.^60,61^ The limited efficacy of targeting only one out of several altered proteins has been discussed by others, and one conclusion has been that probably the most effective strategy would be to target more than one of the culprit proteins.^62–65^

One major drawback with all of the above is the lack of studies including data on PET, CSF and blood biomarker outcomes from subjects who have also undergone neuropathological assessment after death. Autopsies are rarely carried out nowadays, and parallel neuropathological examinations are even more uncommon.^66^ Only a few percent of brains from demented individuals undergo PM assessment, and these examinations are primarily carried out in cases with an unusual clinical phenotype or in younger subjects with a suspected genetic cause. There seems to be an assumption that the neuropathology in most demented subjects is straightforward, and thus PM assessment is not required; but is it? This represents a major pitfall, as we draw our conclusions based on the presumption that the underlying brain pathology corresponds to what is theoretically expected.

Based on the above, given the crudeness of current biological diagnostic markers and the frequent occurrence of concomitant pathology, it is important for the community to recognize that only a small proportion of aged subjects are likely to benefit from the currently available treatments directed at one altered protein. A decision should therefore be made regarding who should receive such treatment in order to avoid generating disappointing outcomes. Should we treat only younger individuals, many of whom are likely to have genetically driven forms of the disease, or should we also treat older individuals, acknowledging that, based on brain pathology, only around 30% are likely to benefit? Furthermore, it is essential that the brains of treated subjects are assessed postmortem.

### Study limitations

The methods used and assessment strategies implemented were standardized. The case selection was biased as only 12% of all deceased are referred for an autopsy in our region and the neuropathological assessment was carried out on some 30% of them. The clinical groups were defined crudely based on the information obtained from the referral chart. The clinical assessment was either a simple one primarily based on interview of patient or relatives or more thorough including imaging and CSF analysis. Furthermore, we did not have the timelapse from the clinical assessment to the time of death. All the above certainly influence the outcome, pathological alterations in relation to clinical presentation, and needs to be considered.

### Conclusion

Neuropathological assessments of 1825 brains revealed that disease-related altered proteins are frequently observed in the aging population. Only 23 subjects, whose ages at death ranged from 50 to 102 years, showed no detectable protein alterations. The most common protein alteration was the combination of HPτ and Aβ, seen in 64% of the whole cohort, 58% of non-demented individuals, and 86% of those with dementia. The incidence of this pathology increased significantly with age and was often accompanied by additional alterations, particularly TDP-43 and/or αS. These findings suggest that, with sensitive biological diagnostic markers, a substantial number of individuals would have been identified as being at risk of developing AD. Based on current knowledge, the available biological diagnostic markers would have identified those with intermediate or high levels of ADNC. In our cohort of 1825 cases, 506 subjects (28% of the whole cohort) fulfilled these criteria—73% of all 372 demented subjects and 16% of all 1453 non-demented subjects. Moreover, a high proportion of subjects with intermediate to high levels of ADNC displayed concomitant pathologies: only 26% of the 235 non-demented subjects and 21% of the 271 demented subjects displayed pure ADNC. Thus, although biological diagnostic markers can confirm the presence of ADNC in the brain, they cannot reliably provide a “definite” diagnosis regarding the cause of cognitive impairment. Based on the above, it is difficult to identify which individuals would genuinely benefit from single target therapies. It is therefore important to facilitate PM neuropathological assessments, particularly in those who have undergone pre-mortem assessment using various biological diagnostic markers. This is imperative to obtain a reliable understanding of the correlation between pre-mortem biomarker findings and PM neuropathological alterations. Moreover, it is crucial to facilitate PM neuropathological assessment in individuals who have participated in clinical trials. Only with this approach can we, over time, move from hypothesis to evidence regarding the underlying brain alterations. This is essential for advancing a fully effective precision medicine in the field.
